# Anaesthetic Management for Cataract Surgery in VACTERL Syndrome Case Report

**Published:** 2009-02

**Authors:** Sonal S Khatavkar, S R Jagtap

**Affiliations:** 1 Lecturer, Department of Anaesthesiology, Dr. D. Y. Patil Medical Collage & Hospital, Nerul, Navi-Mumbai 400706; 2 Prof. & Head, Department of Anaesthesiology, Dr. D. Y. Patil Medical Collage & Hospital, Nerul, Navi-Mumbai 400706

**Keywords:** VACTERL Syndrome, Anaesthesia (Paediatric), Surgery (Cataract)

## Abstract

**Summary:**

Eight year old girl, weighing 14 kg with VACTERL syndrome V: Vertebral anomalies, A: Anal malformation, C: Cardiovascular defect, TE: Tracheal and esophageal malformation, R: Renal agenesis, L: Limb anomalies. underwent cataract surgery under general anaesthesia. She had multiple congenital anomalies like esophageal atresia, imperforate anus (corrected), single kidney & radial aplasia. Anticipating problems of gastro-esophageal reflux & chronic renal failure, successful management was done.

## Introduction

VATER Association is a set of congenital malformations occurring in different combinations (at least two). These present as major functional impairment and appear during first year of life.^1^

VATER is anacronym or abbreviation representing the first letter of each feature in association. Because of some non-causal malformations whether this pattern is sequence or syndrome is still unknown. For such situations the word association has been coined. The VATER has been now abbreviated as VACTERL. [V: Vertebral anomalies, A: Anal malformation, C: Cardiovascular defect^2^, TE: Tracheal and esophageal malformation, R: Renal agenesis^3^, L: Limb anomalies.]

This malformation predominantly occurs in association and with sporadic presentation in families with-out previous history.^4^

Here we report the anaesthetic management of a rare case of VACTERL syndrome for cataract surgery.

## Case report

Eight year old girl, weighing 14kg presented with diminished vision for six months. Cataract was diagnosed in both the eyes. She was full term normally delivered child, detected to have esophageal atresia & imperforate anus after few hours of birth. She also had absent radius. A single kidney was accidentally detected on sonography.

At 30 hours of life she had undergone feeding gastrostomy, colostomy & esophageal pouch was created. At the age of 1½ years, she had undergone anal pull through surgery & colostomy closure was done. At the age of 2 years, esophageal atresia was corrected by formation of tube of colon anastomosing with stomach; subsequently esophageal pouch was closed. At the age of 6 years she presented with anuria & diagnosed to have renal hypertension and chronic renal failure due to single kidney. She was on hemodialysis initially once a week now twice a week. Prior to the day of surgery, she had undergone hemodialysis. She was on oral amlodepin 5mg BD & Losartan 50mg ½ HS since 2 years. She had deafness since 8 months, using hearing aids and now presented with diminution of vision of both the eyes, diagnosed as bilateral cataract.

**Fig 1:**
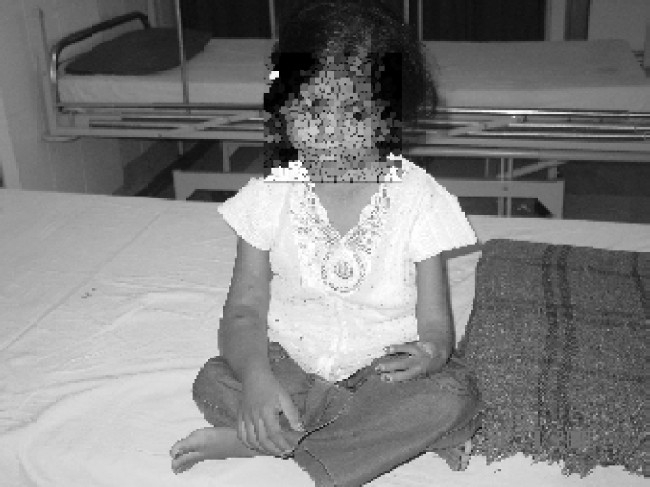
Patient (VACTERL Syndrome)

**Fig 2:**
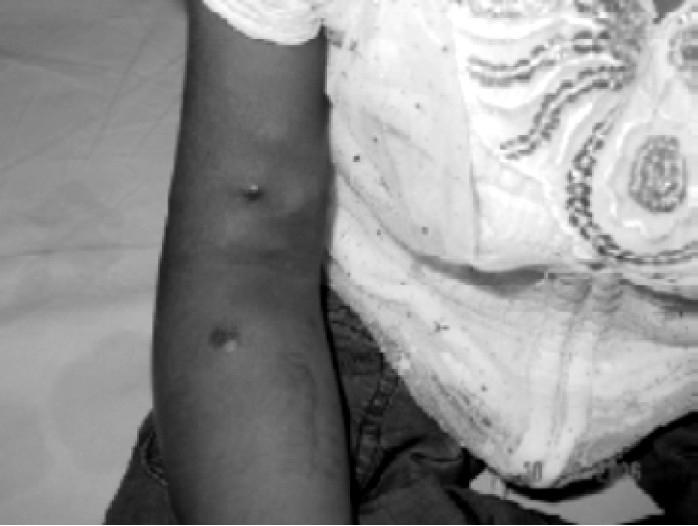
Right hand with AV fistula

**Fig 3:**
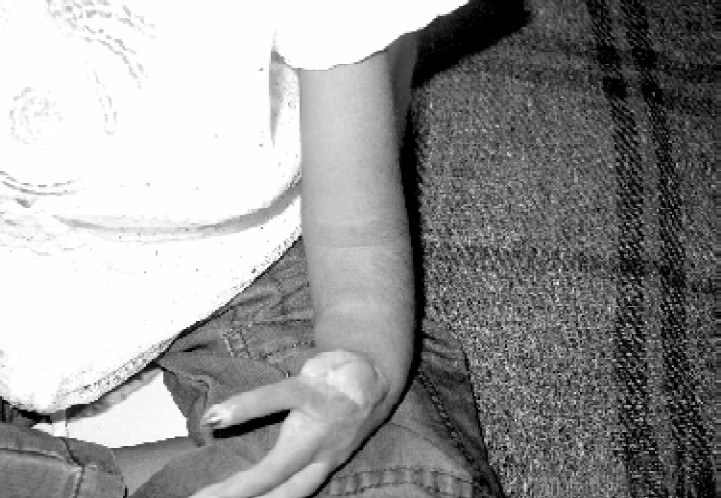
Left hand with radial aplasia

**Fig 4:**
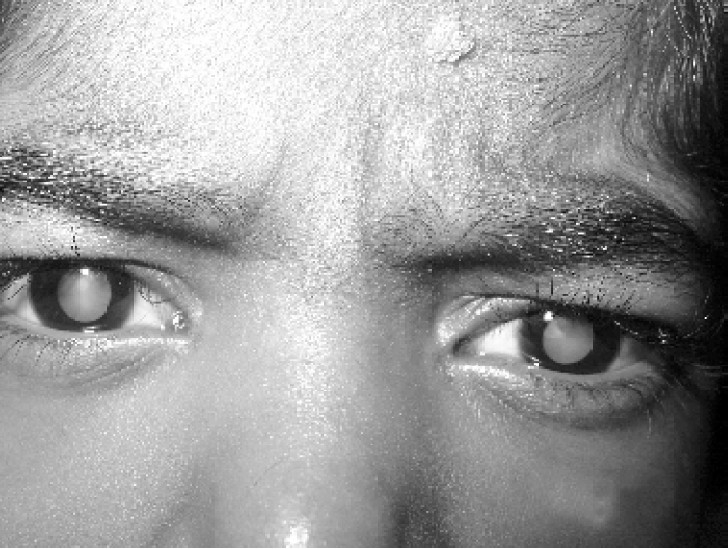
Bilateral cataract

On clinical examination, (Fig. 1–4), she was malnourished, weighing 14 Kg & height of 100cm. She was deaf, non-cooperative and unable to stand due to weakness. Her left arm was short due to Radial aplasia. Right arm & forearm had AV fistula. Her pulse rate was 120/min regular; blood pressure 160/100mmHg measured on left upper arm and body temperature was normal. She was pale, no icterus or cyanosis was observed. Her spine was normal. In cardiovascular system, S1\S2 was normal with soft systolic murmer. On respiratory system examination, airentry was equal on both the sides; there were no foreign sounds. Mallampati classification could not be elicited, as child was un-cooperative.

The blood investigations reported haemoglobin 6.9gm%, blood group B+, haematocrit 19%, random blood sugar 75mg, bleeding time 2 min 5 sec, prothrombin time 13 sec & I.N.R. 1:1. Before dialysis, blood urea was elevated to 70mg/dl, serum creatinine 5.3mg/dl & serum potassium 5.6mmol/L. After dialysis, blood urea was reduced to 15mg/dl, serum creatinine 1.8mg/dl & serum potassium 3.7mmol/L. Her ECG showed sinus tachycardia and in chest X-ray, there was cardiomegaly. On 2-D echo, mild concentric LVH, diastolic dysfunction, ejection fraction 60% was reported.

## Anaesthesia management

Patient was classified as ASA grade IV in view of multiple anomalies and chronic renal failure. Patient was dialysed day prior to the surgery & blood investigations were done. Patient was an operated case of esophageal atresia, in which tube of colon was connected from esophagus to stomach. In this operated patient since esophageal sphincter does not exists, so there is always risk of regurgitation. Anticipating the risk of regurgitation, patient was kept nilper orally for 12hrs. Antacid (Tab. ranitidine 2-4mg.Kg^−1^, 1/4^th^ tablet) and anti-hypertensive drugs were given with sips of water two hrs before surgery. Peripheral IV cannulation with 22g cannula was done, DNS infusion was started. High-risk consent was obtained and patient shifted to operation theatre. Nasogastric tube could not be passed, as child was uncooperative. Cardioscope, pulse oximeter, NIBP were attached to the patient. Glycopyrrolate 60mcg & ondansetron 1mg was given IV. She was preoxygenated with 100% oxygen for 5min, after that midazolam 0.4mg, tramadol 30mg IV were given. Patient was induced with thiopental 100mg IV & atracurium 8mg IV was given. At the time of induction through the transparent mask, reflux of regurgitated material was seen in oral cavity. Patient was still breathing spontaneously as relaxant effect was yet to come & no IPPV was given. Immediately head low was done & suction done with large bore catheter. Under direct laryngoscopy, orotracheal intubation was done with 5 mm I.D. ETT and ventilated with Jackson & Rees circuit with 100% oxygen. Air entry was checked, tube fixed, throat pack kept. Saturation dropped up to 92%, so endotracheal suction was done with saline, through endotracheal tube, no foreign material was retrieved. Hydrocortisone 20mg & dexamethasone 2.8mg IV were given. Saturation improved upto 98%, there were no foreign sounds on chest auscultation. Then patient was ventilated with oxygen (50%), nitrous (50%) & halothane intermittently. Surgery was allowed to start, as patient was stable maintaining saturation 98%-99%. Intra-operatively vitals were stable, all care to avoid hypothermia & fluid over load was taken in view of CRF. Total surgery time was 45min. X-ray chest on table showed clear lung field. After regaining spontaneous respiration, throat pack was removed, suction done & reversed with neostigmine 0.8mg & atropine 0.3mg. Patient was extubated after she was fully awake. Vitals were stable & shifted to PICU for observations. Postoperatively she was kepton antibiotic & humidified oxygen on venti-mask She did not require bronchodilator or nebulization.

## Discussion

VACTERL syndrome can pose problems for an anaesthesia due to multiple anomalies.

In 1975 VATER was expanded to VACTERL^5^ which includes V: Vertebral anomalies 70%, A: Anal anomalies 80%, C: Cardiac problems 53%, TE: Tracheo-esophageal atresia70%, R: Renal anomalies 53% & L: Limb anomalies 65%. Radial defect is commonly detected at birth so it is mandatory^6^ to rule out other anomalies. Sporadic association of specific birth defects^7^ characterizes the VACTERL syndrome. Quan & Smith first developed the term VATER association in 1973. VATER association is genetically considered a polytopic change, thus related to blastogenesis & different from monotopic change related to organogenesis^8^. Although the uncertainty about malformation origin, whether genetic or predisposed^9^, all involved structures are genetically normal. Even parts secondarily affected by them (spinabifida) are genetically normal.^10^

Our patient presented with bilateral cataract. She also had single kidney & radial agenesis. In early childhood patient had undergone correction of esophageal atresia where colon was used for reconstruction of esophagus. The disadvantage of this is colon does not have sphincteric action & motility is also slow. So the risk of gastro-esophageal reflux^11^,^12^ is 50%. Threat to the life can occur during induction due to reflux of regurgitated particles & soiling of lungs parenchyma. With the anticipation of above problem, patient was kept nil perorally for 12h. Head up was done. Pro kinetic were not given in view of extra-pyramidal side effects. Adequate preoxygenation was done. The large bore suction & transparent mask were kept ready. Also vigilant check was kept for food particles regurgitating in mouth. Thus all steps to avoid aspiration of food particle in lungs were done. With this we could avoid soiling of the lungs. But situation may not be the same always. To avoid the risk of aspiration rapid sequence induction with suxamethonium is an ideal technique. Our patient was the case of CRF on dialysis with multiple congenital defects & muscle weakness so we could not use suxamethonium, due to potential risk of hyperkalemia leading to cardiac arrest. In these patients care must be taken to avoid hyperkalemia, fluid overload, hypothermia. Also due care of AV fistula is needed to avoid injury. Abnormalities in bleeding profile & coagulation can lead to intra-ocular bleeding, as the patient was on dialysis. Patient was anaemic, however she had lost her vision so the risk was taken to improve her vision. After explaining the anaesthesia risk to the relatives with informed consent patient was accepted for surgery.

Patient with tracheo-esophageal fistula has susceptibility to respiratory infections due to weakness in tracheal muscles & hyper reactive airways. These patients need active respiratory care with physiotherapy & antibiotics, pre & postoperatively. When the colon is used for anastomosis, since esophageal sphincter does not exists, there is always risk of regurgitation. A silent regurgitation is a real threat to life at induction of anaesthesia even if patient is adequately starved. Hence history of regurgitation even at rest after food should be ruled out pre-operatively, on repeated enquiry relatives came out with this history in our patient. This demands vigilance & extra care to avoid aspiration.

In conclusion the pre-existing anomalies with potential risk of regurgitation in patient with VACTERL syndrome demands careful pre-operative assessment and use of skillful anaesthetic technique to avoid fatal complications.
